# Early adapted physical activity to prevent and manage aromatase inhibitor-induced musculoskeletal pain in breast cancer: protocol for a hybrid effectiveness-implementation randomised controlled trial (APIS)

**DOI:** 10.1136/bmjsem-2025-003179

**Published:** 2026-05-14

**Authors:** Nathalie Piazzon, Elise Verot, Annick Gérard, Amandine Baudot, Florence Carrouel, Marion Cortet

**Affiliations:** 1 Service de Gynécologie-Obstétrique, Hôpital de la Croix Rousse, Hospices Civils de Lyon, Lyon, France; 2 Health Systemic Process (P2S), UR4129, University Claude Bernard Lyon 1, Lyon, France; 3 CIC EC 1408 INSERM Saint-Etienne, Centre Hospitalier Universitaire de Saint-Etienne, Saint-Étienne, Auvergne-Rhône-Alpes, France; 4 CHU de Saint-Étienne, Saint-Etienne, France; 5 LabTAU, INSERM U 1032, University Claude Bernard Lyon 1, Lyon, France

**Keywords:** Oncology, Pain Management, Physical activity, Implementation, Randomised controlled trial

## Abstract

Aromatase inhibitors are the standard adjuvant hormone therapy for postmenopausal women with hormone-sensitive breast cancer. However, nearly half of patients experience aromatase inhibitor-associated musculoskeletal syndrome (AIMSS), predominantly pain, which compromises quality of life and treatment adherence. While adapted physical activity offers proven benefits in oncology, its specific role in preventing or managing AIMSS remains unclear. Moreover, the maintenance of physical activity throughout the care pathway in real-world settings is limited, underscoring the need for hybrid approaches that assess both clinical effectiveness and implementation. In response to these challenges, the primary study aim will be to compare the prevalence of musculoskeletal pain at 6 months of aromatase inhibitor therapy between patients who initiate a personalised, adapted physical activity programme at the beginning of the care pathway and those who receive usual care. Secondary aims will be to (1) assess additional effects of the intervention on physical health, psychosocial well-being and treatment adherence, (2) explore contextual factors influencing programme implementation in routine oncology care and (3) identify potential risk factors for the development of AIMSS.

The APIS study is a hybrid type I effectiveness-implementation randomised controlled trial including 182 postmenopausal women with non-metastatic hormone-sensitive breast cancer.APIS will generate new evidence on the clinical and implementation effectiveness of early personalised adapted physical activity in preventing AIMSS. The hybrid design will support the development of sustainable, patient-centred interventions potentially improving quality of life, adherence to hormone therapy and long-term outcomes in breast cancer survivorship.

Trial registration number

NCT07295457.

**WHAT IS ALREADY KNOWN ON THIS TOPIC**

- Aromatase inhibitors are a standard treatment for hormone-sensitive breast cancer and are frequently associated with musculoskeletal pain (AIMSS), which negatively affects quality of life and treatment adherence. Although physical activity is recommended in oncology, evidence for its specific role in preventing AIMSS is limited, and the implementation of exercise interventions in routine care remains suboptimal.

**WHAT THIS STUDY ADDS**

- To date, the preventive role of early adapted physical activity on the development of aromatase inhibitor-induced musculoskeletal symptoms has not been specifically investigated.
- This study combines the evaluation of clinical effectiveness with an embedded implementation analysis using a hybrid type 1 design.
- It explores the emerging role of advanced practice nurses within an interdisciplinary care pathway to support the integration and sustainability of adapted physical activity in oncology.

**HOW THIS STUDY MIGHT AFFECT RESEARCH, PRACTICE OR POLICY**

- This study will provide a more comprehensive understanding of the effects of early adapted physical activity on aromatase inhibitor-induced musculoskeletal symptoms and generate insight into interdisciplinary implementation processes within routine oncology care.

## Background

 Breast cancer is the most frequently diagnosed cancer in women worldwide, responsible for 666 103 deaths in 2022. Approximately 80% of these cases are hormone-sensitive.^1^ In postmenopausal women with non-metastatic hormone-sensitive breast cancer, the standard treatment regimen includes surgery followed by hormone therapy. Depending on tumour characteristics, this approach may be complemented by chemotherapy and/or radiotherapy. Hormone therapy is initiated at the end of primary treatment and continues during the survivorship care period in more than 98.5% of cases.^2^ Aromatase inhibitors (AI) are the standard adjuvant hormone therapy for postmenopausal women with hormone-sensitive breast cancer, effectively reducing recurrence and mortality rates.^1^

AIs can cause AI-associated musculoskeletal syndrome (AIMSS) that affects nearly half of treated women and negatively impacts their quality of life and treatment adherence. AIMSS involves musculoskeletal symptoms such as arthralgia, myalgia and tendinopathy, with pain being the most common and significant symptom.^3^ The physiopathology and predictive factors of AIMSS remain unclear. The physiopathology and predictive factors behind AIMSS remain unclear. They could be linked to oestrogen deprivation, which is known to play a role in maintaining joint and bone health by promoting cartilage regeneration and reducing inflammation.^3^ Both pharmacological and non-pharmacological strategies have been investigated for managing AIMSS, but evidence on accessible and effective therapies for its prevention or treatment remains limited.^4^

Supportive care, particularly regular physical activity (PA), offers many benefits for people with cancer.^5^ Most research focused on women with breast cancer. The practice of PA during and after medical treatment is associated with reduced fatigue,^6^ improved quality of life,^7^ lower recurrence risk and better survival rate.^8^ Schmitz *et al*^9^ emphasise the importance of initiating PA early in the oncology care pathway to prevent the progressive impact of inactivity on physical function. This concern is reinforced by recent evidence showing that skeletal muscle deconditioning can appear within the first weeks of chemotherapy in patients with breast cancer.^10^ Together, these findings have informed the development of international guidelines recommending the integration of PA into oncology care pathways.^5^

To translate these benefits into clinical practice, adapted physical activity (APA) enables trained professionals to support patients in developing individualised PA programmes tailored to their daily lives and care pathways. According to the International Federation of APA, APA is ‘directed toward persons who require adaptation for participation in the context of PA. This involves individualising instruction, matching personal strengths and interests with appropriate activities and adapting environments to promote full participation in PA’.^11^

In this context, the early implementation of APA, initiated at the start of the care pathway, could help prevent or alleviate treatment-related side effects, including musculoskeletal symptoms such as AIMSS. However, to date, no study has specifically investigated the role of PA in preventing AIMSS and current evidence remains insufficient to support exercise-based recommendations for its management.^12^

In real-world settings, many patients with cancer struggle to initiate and maintain regular PA during and after treatment, with adherence to recommended guidelines ranging from only 18% to 41%.^13^ Moreover, fewer than 50% of evidence-based PA interventions in oncology are implemented into routine clinical practice.^14^ This gap highlights the complex, multifactorial barriers to implementation that remain underexplored in exercise oncology. To address this issue, hybrid research designs are increasingly recommended, as they enable the simultaneous evaluation of clinical effectiveness and real-world implementation conditions.^15^

### Study objectives

The primary study aim is to compare the prevalence of musculoskeletal pain after 6 months of AI therapy between patients who started a personalised APA programme at the start of their care pathway and those receiving usual care.

The secondary objective is to explore contextual factors influencing the implementation of the APA programme in real-world oncology care, including perceived barriers and facilitators, based on quantitative and qualitative data collected from both patients and healthcare professionals.

## Method

### Study design

**Figure 1:**
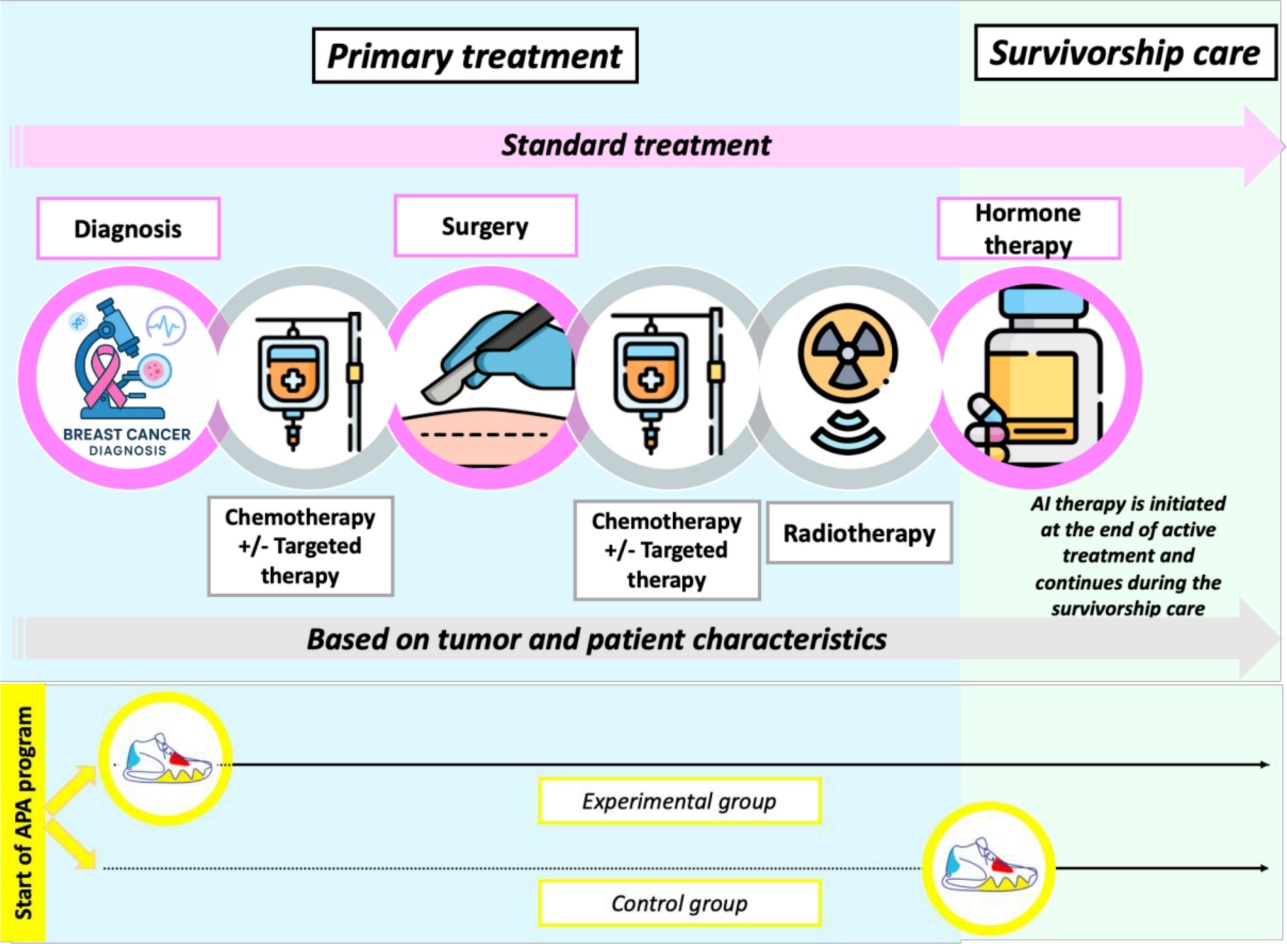
Standard treatment pathway for postmenopausal women with non-metastatic hormone-sensitive breast cancer, including timing of APA programme initiation in the experimental and control groups.AI: Aromatse Inhibitors; APA: Adapted Physical Activity.

The APIS trial is a type 1 hybrid randomised controlled trial (RCT), evaluating the effectiveness of the intervention, while also collecting data on key implementation factors in real-world conditions.^16^ This study is designed as an open-label, two-arm, parallel, multicentre, RCT. Patients in the experimental group will begin a personalised APA programme at the start of primary treatment (surgery or neoadjuvant chemotherapy), in parallel with their usual treatment, as illustrated in figure 1. Patients in the control group will receive standard care, with the structured APA programme initiated at the beginning of the AI therapy, namely at the end of primary treatment. In the absence of standardised real-world data on when to initiate APA in postmenopausal women with hormone-sensitive breast cancer, the timing chosen for the control group reflects current local practice. Both groups will have no restrictions on autonomous PA during their care pathway.

**Figure 2:**
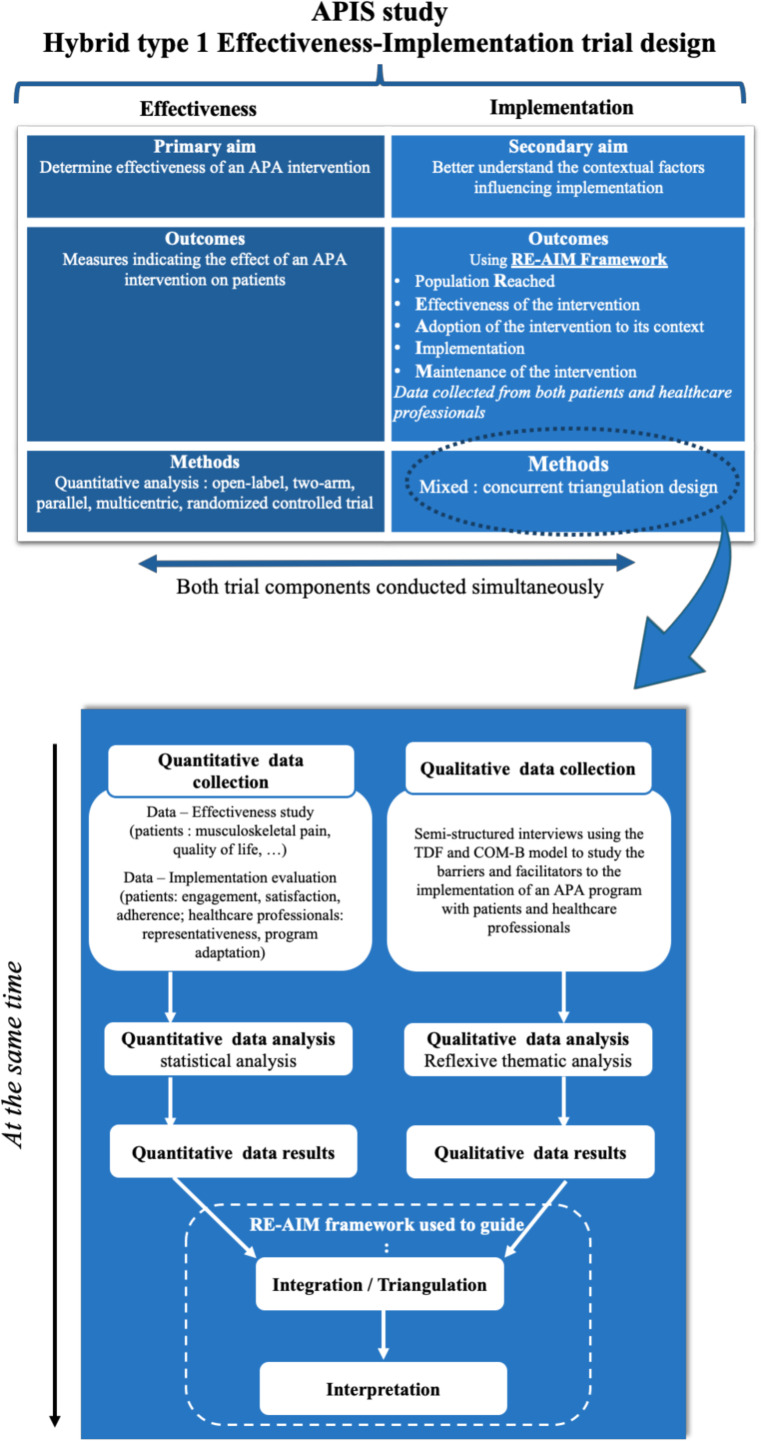
APIS study methodology.APA :adapted physical activity; COM-B :Capability, Opportunity, Motivation – Behaviour; RE-AIM : Reach, Effectiveness, Adoption, Implementation and Maintenance; TDF : Theoretical Domains Framework.

In accordance with its secondary objective, the APIS trial also incorporates an embedded implementation evaluation. A concurrent mixed-methods design with methodological triangulation will be used. Quantitative and qualitative data will be collected in parallel, analysed separately and then integrated to explore implementation outcomes.^17^ Patients and healthcare professionals involved in the care of patients with breast cancer will be invited to participate, to capture implementation-related experiences, perceptions and contextual information. The study design is shown in figure 2.

The APIS protocol was developed in accordance with the Standard Protocol Items for Randomised Trials (SPIRIT 2025) guidelines for interventional studies, and the intervention is described using the Template for Intervention Description and Replication - Rehab checklist to ensure replicability.

### Study setting

This study will be conducted in two French hospitals: the gynaecological surgery departments of the Saint-Etienne University Hospital and the Lyon University Hospital (Hospices Civils de Lyon).

### Study population

The population will include patients and healthcare professionals.

#### Patients

The inclusion criteria will be: (1) female, (2) aged between 18–75 years, (3) diagnosed with non-metastatic breast cancer, (4) hormone-sensitive breast cancer, (5) indication for treatment with AI, (6) affiliated with a social security scheme, (7) provided signed informed consent.

The exclusion criteria will be: (1) individuals deprived of liberty by a judicial or administrative decision, (2) undergoing psychiatric care for an unstable condition, (3) subject to a legal protection measure (guardianship or curatorship), (4) unable to receive sufficient information due to cognitive disorders or insufficient proficiency in the French language, (5) participating in another interventional study with an ongoing exclusion period at pre-inclusion, (6) presenting absolute contraindications to PA (HAS, 2019, p.61), (7) presenting a locomotor system condition that makes cycling impossible.

#### Healthcare professionals

The inclusion criteria will be: (1) individuals working at the Saint-Etienne University Hospital and the Lyon University Hospital as a surgeon, oncologist, radiotherapist, sports medicine physician, APA instructor, physiotherapist, head nurse, care coordinator nurse or advanced practice nurse, (2) caring for patients treated with AI for non-metastatic breast cancer, (3) having signed the informed consent form.

### Assignment of interventions

Participants will be randomised using Ennov Clinical software. Randomisation will be stratified. The study centre will use a computer-generated pseudo-randomised list prepared in advance by a biostatistician. Given the behavioural nature of the intervention, blinding will not be possible.

### Intervention

The APA programme, delivered by the sports medicine department, will consist of structured exercises supervised by APA professionals and will be identical for all patients enrolled. The only difference between the two groups is the timing of the intervention within the care pathway: either initiated at the diagnosis of cancer or at the introduction of AI. The APA programme will begin, after a medical evaluation, with a 12-week supervised programme. Each week, the patient will have two 1 hour sessions supervised by a physiotherapist and/or an APA instructor. At the end of the initial 12-week APA programme, the sports medicine physician assesses the patient’s progress and goal attainment to decide whether to renew the programme, refer the patient to a supervised community-based PA, or guide her toward self-directed activity.

After 12 weeks, a new medical consultation will be scheduled, and a new renewal or adjustment phase will begin until the end of the trial.

The detailed APA programme is provided in online supplemental file 1.

In addition to this personalised programme, the interdisciplinary care team will encourage patients to remain physically active throughout the rest of the week, according to their individual abilities. The goal will be for patients to develop specific skills that will support long-term lifestyle changes.

### Study design and assessment time

**Figure 3:**
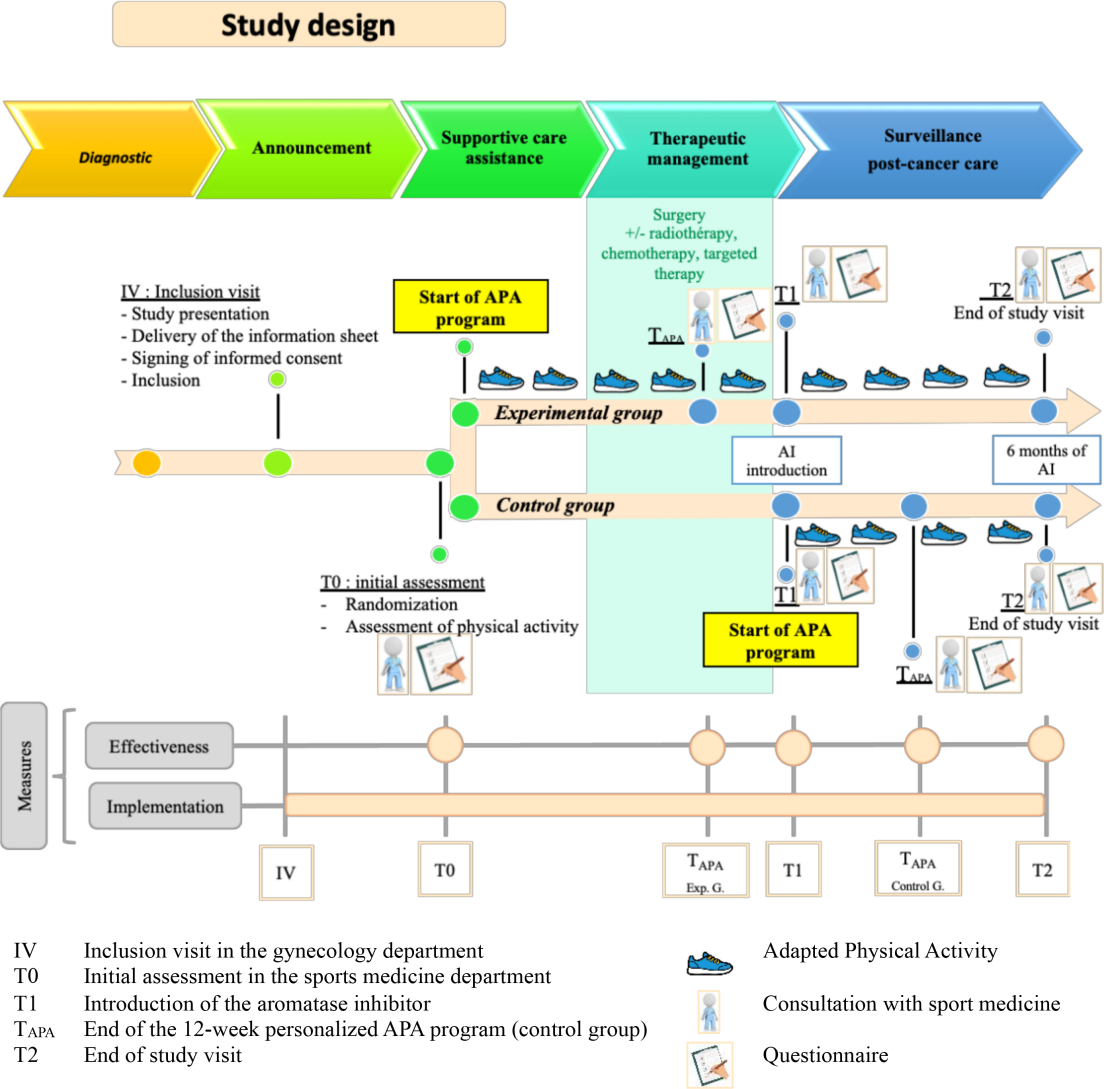
Recruitment and study procedures. AI :Aromatase Inhibitors; APA :Adapted Physical Activity.

Figure 3 illustrates the study design aligned with the clinical care pathway, including the timing of randomisation, the APA intervention and data collection time points. Data collection will be structured around standardised key events in the treatment pathway: T0 (during the pre-therapeutic phase), T1 (at the initiation of the AI), T_APA_ (at the end of the 12-week personalised APA programme) and T2 (6 months after the initiation of the AI). The assessment times will be defined by the stages of the care pathway, rather than fixed calendar times, to account for variability in treatment protocols. The interval between T0 and T1 may range from 1 month for patients treated with mastectomy alone, to up to 7 months for those treated with surgery, chemotherapy (with or without targeted therapy) and radiotherapy. The interval between T1 and T2 is fixed at 6 months.

### Outcomes

#### Primary outcome

The primary outcome will be the score for the most intense musculoskeletal pain, assessed with the Nordic-style questionnaire, 6 months after the introduction of the AI (T2).

#### Secondary outcomes: effectiveness aim

Secondary outcomes will evaluate the effects of the intervention on physical health and psychosocial well-being by assessing global pain, cognitive problems, physical condition, body composition and nutritional status, indicators of metabolic syndrome, heart rate variability, therapeutic adherence to the AI and adverse events related to the intervention. These outcomes will be assessed at four time points (T0, T1, T_APA_, T2) using validated questionnaires, clinical examinations and functional tests.

#### Secondary outcomes: implementation aim

The implementation of the APA programme will be evaluated using the Reach, Effectiveness, Adoption, Implementation and Maintenance (RE-AIM) framework, which is designed to assess both the public health impact of interventions and the processes underlying their implementation.^18^ All five RE-AIM dimensions will be examined to capture key factors influencing real-world applicability, using a combination of quantitative and qualitative data collected from participants and healthcare professionals throughout the study.

#### Secondary outcomes: risk factors for the development of AIMSS

The APIS study will investigate potential risk factors for the development of AIMSS by examining associations between clinical, demographic, treatment-related and psychosocial variables and the occurrence of musculoskeletal symptoms.

### Participant timeline

The assessment schedule for both groups is presented in table 1.

**Table 1:** Schedule of study visits and assessments

	IV	T0	T1	T_APA_	T2
**Enrolment**					
Review of inclusion/exclusion criteria	**X**				
Study presentation/delivery of the participant information sheet	**X**				
Signature-informed consent	**X**				
**Randomisation**					
Group allocation		**X**			
**Intervention**					
Start of personalised APA protocol, **experimental group**		**X**			
Start of personalised APA protocol, **control group**			**X**		
**Assessments**					
Initial clinical assessments*	**X**				
Clinical assessments†		**X**	**X**	**X**	**X**
Anthropometric measurements‡		**X**	**X**	**X**	**X**
6MWT		**X**	**X**	**X**	**X**
Hand grip test		**X**	**X**	**X**	**X**
Pulmonary function test§§		**X**			
Cardiopulmonary exercise testing¶¶		**X**			**X**
Blood test****		**X**	**X**		X*
24-hour Holter ECG monitoring††††		**X**			**X**
Self-Report Questionnaires‡‡		**X**	**X**	**X**	**X**
Self-directed physical activity follow-up: email or paper logbook§§		**X**	**X**	**X**	**X**
Data collected/implementation		**X**	**X**	**X**	**X**
Data collected/risk factors for the development of AIMSS			**X**		
Semi-structured interview					**X**
Monitoring of adverse events			**X**	**X**	**X**
Monitoring of APA-related adverse events			**X** EG	**X**	**X**

* Initial clinical assessments performed by the surgeon or APN: clinical examination, identification of barriers and facilitators to regular physical activity during the medical interview and assessment of absolute and temporary contraindications to adapted physical activity.

† Clinical assessments performed by the sports medicine physician.

‡ Anthropometric measurements: weight, height, BMI, waist circumference, four skinfolds.

§ Pulmonary function test: FEV1, PEF, VT, VC, RV, TLC, DLCO correction.

¶ Cardiopulmonary exercise testing: VO₂ peak, VO₂ max, maximal heart rate, VO₂ at ventilatory threshold -VT1-, cardiac output at VT1, heart rate at VT1, blood pressure, oxygen saturation.

** Blood test: lipid profile at V0 and V1 (V2 if abnormal value at V1), fasting insulin and fasting blood glucose at V0 (V1 if abnormal value at V0 and V2 if persistent abnormal value at V1), C-reactive protein at V0 (V1 and V2 if inflammation is suspected).

†† 24-hour Holter ECG monitoring: total spectral power.

‡‡ Self-Report Questionnaires (cf. table 2).

§§ Self-directed physical activity follow-up: type, intensity (Borg scale), duration, frequency of physical activity and adverse effects of PA.

.AIMSS, aromatase inhibitor-induced musculoskeletal symptoms; APA, adapted physical activity ; APN, Advanced Practice Nurse; BMI, body mass index; CG, control group; CRP, C-reactive protein; D, day; DLCO, diffusing capacity of the lung for carbon monoxide; EG, experimental group; FEV1, forced expiratory volume in 1 s; HOMA-IR, Homeostatic Model Assessment for Insulin Resistance; IV, inclusion visit; M, month; MWT, 6-minute walk test; PA, physical activity; PEF, peak expiratory flow; RV, residual volume; T0, pre-therapeutic phase; T1, initiation of the aromatase inhibitors; T2, 6 months after the initiation of the aromatase inhibitors; T_APA_, end of the 12-week APA programme; TLC, total lung capacity; VC, vital capacity; VO₂ max, maximal oxygen consumption; VO₂ peak, peak oxygen consumption; VT, tidal volume.

**Table 2:** Summary of secondary outcomes and data collection methods for the effectiveness aim

Outcome domain	Outcome measure	Data collection	Assessment modality
Global pain*	Musculoskeletal pain	Musculoskeletal pain intensity and localisation	Nordic-style questionnaire†
	Overall pain	Self-rated overall pain	Numerical Rating Scale†
	Quality of life	Quality of life score	FACT-B†, FACT-ES†
	Fatigue	Fatigue score	FACIT – Fatigue†
	Anxiety	Anxiety and depression scores	HADS†
**Cognitive function**		Perceived cognitive impairments	FACT-Cog†
**Physical condition**	Respiratory function	FEV1, PEF, VT, VC, RV, TLC, DLCO	Pulmonary function tests
	Cardiac function	VO₂ peak, VO₂ max, maximal heart rate, VO₂ at VT1, cardiac output at VT1, heart rate at VT1, blood pressure, oxygen saturation	Cardiopulmonary exercise test
	Cardiorespiratory endurance	Distance walked in 6 minutes	6-minute walk test
	Muscular strength	Isometric grip strength	Handgrip strength test
	PA level	Habitual levels of PA	Marshall questionnaire†, IPAQ†
**Body composition**	Anthropometric measurements	Body weight, height, body mass index, waist circumference and 4-site skinfold	Anthropometric assessment
	Malnutrition	MUST score	MUST†
**Evolution of a metabolic syndrome**		Waist circumference	Anthropometric assessment
		Blood pressure	Blood pressure measurement
		Lipid profile, assessment of insulin resistance and inflammatory status	Blood tests: lipid profile, HOMA-IR, C-reactive protein
**HRV‡**		HRV parameters	24-hour Holter ECG recording
**Therapeutic adherence to the AI**		Therapeutic adherence to the AI	Girerd self-assessment questionnaire†
**Adverse events related to APA**		Adverse events related to APA sessions	Participant self-report

* The evaluation of AIMSS requires a structured and multidimensional approach. It will include a targeted assessment of musculoskeletal pain, a self-report of overall perceived pain and an analysis of its psychosocial impact.

† Self-Report Questionnaires.

‡ Heart rate variability is a non-invasive indicator of autonomic cardiac regulation, reflecting the balance between sympathetic and parasympathetic activity. A decrease in HRV has been associated with increased clinical vulnerability in oncology. Several studies have reported associations between altered HRV and clinical parameters such as fatigue, pain and tumour progression.

.AI, aromatase inhibitor; AIMSS, AI-associated musculoskeletal syndrome; APA, adapted physical activity; DLCO, diffusing capacity of the lung for carbon monoxide; FACIT-Fatigue, Functional Assessment of Chronic Illness Therapy – Fatigue; FACT-B, Functional Assessment of Cancer Therapy – Breast; FACT-Cog, Functional Assessment of Cancer Therapy - Cognitive Function; FACT-ES, Functional Assessment of Cancer Therapy - Endocrine Symptoms; FEV1, forced expiratory volume in 1 s; HADS, Hospital Anxiety and Depression Scale; HOMA-IR, Homeostatic Model Assessment for Insulin Resistance; HRV, heart rate variability; IPAQ, International Physical Activity Questionnaire; MUST, Malnutrition Universal Screening Tool; MUST, Malnutrition Universal Screening Tool; PA, physical activity; PEF, peak expiratory flow; RV, residual volume; TLC, total lung capacity; VC, vital capacity; VO_2_ max, maximal oxygen consumption; VO_2_ peak, peak oxygen consumption; VT1, ventilatory threshold 1; VT, tidal volume.

#### Screening: inclusion visit

Eligible patients will be identified either during the cancer diagnosis disclosure consultation with the surgeon or during the consultation with an Advanced Practice Nurse (APN) as part of the pre-therapeutic care pathway in the gynaecological surgery. They will provide written informed consent after receiving comprehensive information about the study and being given adequate time to consider their participation.

#### Initial assessment/randomisation (T0)

A clinical and paraclinical examination by the multidisciplinary sports medicine team during a day care hospital visit, before any therapeutic intervention, will include clinical data, cardiopulmonary exercise testing, pulmonary function testing, 24-hour Holter ECG monitoring, additional examinations and Self-Report Questionnaires. Patients will also be instructed on daily PA self-monitoring via an electronic or paper logbook. The estimated completion time is 2 min per day.

They will be randomised into two groups.

#### Follow-up visit at the initiation of AI (T1)

At the start of AI therapy, all patients will undergo a clinical evaluation, functional tests, adverse event monitoring and Self-Report Questionnaires. This visit will occur in the sports medicine department and be conducted by a sports medicine physician.

For the control group, the personalised APA programme will start at this stage.

#### Follow-up visit at the end of the 12-week personalised APA programme (T_APA_)

T_APA_ will include evaluations similar to those performed at T1.

#### End-of-study visit (T2)

The end of the study will be defined as 6 months after the initiation of AI therapy, which corresponds to the median time point at which both the peak of AIMSS symptoms and treatment discontinuation due to AIMSS are reported in the literature.^19^ This assessment will follow the same procedure as T1 and T_APA_, with the addition of cardiopulmonary exercise testing and 24-hour Holter ECG monitoring.

Semi-structured individual interviews will be conducted by two researchers with patients and healthcare professionals.

### Sample size

#### Sample size for the primary outcome

The expected prevalence of AIMSS in the standard care group is approximately 40%. We hypothesise a 50% reduction (20%) in the experimental group with early implementation of APA.^20^ Considering an alpha risk of 5%, a power of 80% and a two-sided hypothesis, 82 patients per arm will be required. Assuming 10% unusable data, a total of 182 patients will be included.

Sample size for the qualitative component of the mixed-methods study (secondary implementation aim).

The principle of theoretical saturation will guide the sample sizes of the two groups (patients and healthcare professionals), once the collected data are sufficient to explain the phenomenon. Data collection, conducted progressively and in parallel with analysis, will define the final sample size.^21^

### Recruitment

#### Recruitment for quantitative outcomes

The estimated pool of eligible patients is 240 patients at the gynaecological surgery departments at the Saint-Etienne University Hospital and 360 at the gynaecological surgery departments at the Lyon University Hospital over the full 24-month inclusion period. The APIS study will recruit 72 women at the Saint-Etienne Hospital and 110 women at the Lyon Hospital, for a total of 182 subjects.

#### Recruitment for the qualitative component

A purposive sampling strategy based on the maximum variation method will be used to ensure a diverse representation of real-life clinical situations.^22^ This approach will be designed to capture a wide range of perspectives and to identify shared patterns across heterogeneous experiences.

The patient sample will include individuals from both the experimental and control groups, enabling a comprehensive understanding of the intervention’s implementation across varying contexts.

### Data collection

All functional tests and measurements will be performed as part of routine care in a sports medicine department by professionals trained to ensure the reliability, reproducibility and standardisation of the assessments.

#### Primary data collection

The prevalence of musculoskeletal pain will be assessed using the Nordic-style questionnaire, a validated tool for detecting musculoskeletal disorders. It consists of closed-ended questions and can be used as a self-reported questionnaire.

#### Secondary data collection: effectiveness aim

Table 2 presents the outcome domains, specific measures and data collection modalities for the secondary outcomes evaluated as part of the hybrid type 1 trial’s effectiveness aim.

Validated questionnaires and functional tests were selected based on their relevance and prior use in oncology and exercise studies. Full references are available in the online supplemental file 2.

### Secondary data collection: implementation aim

Quantitative data will be collected through questionnaires and will include indicators such as participation rates, programme adherence, patient satisfaction and the representativeness of both patients and healthcare professionals relative to the target population.

The qualitative analysis will be conducted through semi-structured interviews using the Theoretical Domains Framework (TDF) and COM-B Model (Capability, Opportunity, Motivation – Behaviour).^23^ The TDF will help identify and describe barriers and facilitators to APA programme implementation from the perspective of patients and healthcare professionals.^24^

Table 3 summarises the corresponding indicators and data sources for each RE-AIM component.

**Table 3:** RE-AIM framework: definitions and data collection methods in the APIS study

RE-AIM dimension	Definition	Data collection
Reach	Proportion and representativeness of individuals in the target population who participated in the intervention	Percentage of patients who completed all stages of the study, dropout rate at each key stage of the study and reasons for dropout. Comparison of socio-demographic and clinical characteristics of participants (age, treatment, etc) with those of the target population.
Effectiveness	Impact of the intervention on individual outcomes, including benefits, adverse effects and subgroup variability	Primary and secondary outcomes are defined in the effectiveness study.
Adoption	Engagement of individual healthcare professionals in delivering the intervention, in line with the exploratory focus of hybrid type 1 trials	Proportion of healthcare professionals involved in the delivery of the APA programme, by specialty, relative to the total number of eligible professionals at each participating site. Semi-structured interviews with healthcare professionals exploring contextual and behavioural determinants of adoption, such as professional identity, beliefs about capabilities, motivation and perceived organisational barriers or facilitators.
Implementation	Fidelity of intervention	Frequency: Average number of PA sessions performed per week (self-reported and measured within the APA programme). Intensity: Intensity level of PA sessions measured by a perceived exertion scale (Borg scale) and objective data. Time: Average duration of sessions (in minutes). Type: Proportion of sessions complying with the type of exercise recommended by guidelines. Programme-level adaptations (eg, changes in session format, scheduling or delivery methods) will be documented, categorised (structural, organisational or patient-related) and analysed in relation to their impact on ongoing participation. Patient-reported implementation outcomes: motivation* (EMAPS scale) and satisfaction/APA programme (5-point Likert scale). Qualitative data on barriers and facilitators, necessary adaptations and rationale for maintaining or modifying the APA intervention. They will be collected through semi-structured interviews with both patients and healthcare professionals.
Maintenance	Sustainability of the intervention at both the individual and professional levels	Reassessment of FITT indicators at T2. Maintenance will be assessed at T2 to evaluate the sustainability of behaviour and practice changes beyond the APA intervention period. Data on PA levels obtained from logbooks and self-reported questionnaires at T2. Qualitative data on barriers and facilitators to long-term integration of PA into daily life and clinical practice.

* Motivation will be measured using the Échelle de Motivation pour l’Activité Physique à des fins de Santé (EMAPS), a French-language validated tool based on self-determination theory.

APA, adapted physical activity; FITT, Frequency, Intensity, Time, and Type; PA, physical activity.

#### Secondary data collection: risk factors for the development of AIMSS

To investigate potential risk factors for the development of AIMSS, the following data will be collected: clinical, demographic, pathological and therapeutic variables; beliefs and perceptions in relation to hormone therapy will be assessed using the BMQ-AET (Beliefs about Medicines Questionnaire - Adjuvant Endocrine Therapy); and pain catastrophising will be measured using the Pain Catastrophising Scale.

### Data management

#### Quantitative data management

Quantitative data will be collected from several sources: medical records, Self-Report Questionnaires, PA logbooks (paper or email-based) and the results of paraclinical examinations.

They will be entered as they are collected, in each centre, either by authorised research professionals (investigator or team members listed in the delegation of tasks), directly into the electronic case report form, via their personal identifiers and in compliance with data protection regulations, or directly by the patients themselves via the secure Ennov Clinical platform (Ennov, Paris, France).

All data will be pseudonymised using a unique identification code. Automatic validation checks will be applied during data entry, and the sponsor’s clinical research unit will conduct regular monitoring. Any discrepancies or protocol deviations will be documented and communicated to investigators. The trial complies with International Council for Harmonisation Good Clinical Practice and applicable national data protection laws.

#### Qualitative data management

Two distinct semi-structured interview guides, one for patients and one for healthcare professionals, were developed based on the 14 domains of the TDF, following the methodology proposed by Cane *et al*.^25^ Each domain was explored through open-ended questions, supplemented by follow-up prompts as needed to elicit detailed responses.^24^

The guides were reviewed for content validity by two oncology healthcare professionals and a methodological expert to ensure their appropriateness for the target populations. Pilot testing was conducted with a patient partner and a healthcare professional, resulting in refinements to the clarity and wording of the interview items.

Interviews will be conducted at time point T2 by a doctoral candidate (NP) and a patient-researcher (AG), both of whom are trained in qualitative research methods. Each interview will last approximately 25–35 min and will be conducted in a single videoconferencing session. All participants will receive comprehensive written and verbal information about the study, including its objectives, interview procedures and data management protocols. With informed consent, interviews will be audio-recorded using a digital device, securely transferred to a password-protected computer and subsequently deleted from the recording device. Each interview will be transcribed verbatim and pseudonymised. NVivo V.15 software (QSR International) will be used to organise and analyse the data.

### Data analysis

#### Quantitative data analysis

The statistical analysis will be performed on the intention-to-treat population.

For the primary objective, the maximum intensity of musculoskeletal pain at 6 months (T2) will be compared between groups using parametric (eg, t-test) or non-parametric (eg, Wilcoxon-Mann-Whitney) tests, depending on data distribution.

Secondary objectives include evaluating the intervention’s effects on outcomes such as physical health, psychosocial well-being and treatment adherence. Analyses will include: (1) between-group comparisons at specific time points (T0, T1, T_APA_, T2), (2) between-group comparisons of change over time (eg, T0–T1, T1–T2) and (3) within-group longitudinal changes across time points.

Univariate and multivariate logistic regression models will explore factors associated with the occurrence of AIMSS. Qualitative variables will be compared using χ^2^ or Fisher’s exact test, as appropriate.

All analyses will be two-sided with a significance threshold of p<0.05. Missing data will be handled using appropriate statistical methods. Analyses will be conducted using R software (R Foundation for Statistical Computing, Vienna, Austria).

#### Qualitative data analysis

Data will be analysed using a combined deductive and inductive approach to ensure rigour, transparency and reproducibility.^22^ Transcripts will first be deductively coded using the 14 domains of the TDF and COM-B model.

Second, an inductive thematic analysis, following Braun and Clarke’s six-phase methodology,^26^ will be conducted to capture themes not represented within the TDF.

Finally, inductive and deductive codes will be integrated to develop interpretative themes and subthemes, each labelled with a clear, representative title.

## Discussion

The APIS study evaluates the effectiveness of an APA programme initiated early in the oncology care pathway, while also analysing the contextual factors that influence its implementation in routine practice. It further contributes to a better understanding of the risk factors for AIMSS, to identify the most vulnerable patients and support treatment adherence.

### Effectiveness and implementation: justification for a hybrid protocol

This study adopts a type 1 hybrid effectiveness–implementation design, an approach increasingly recommended given the complex, multilevel barriers to integrating PA into routine cancer care. This dual ambition implies the combined use of quantitative and qualitative methods, applied in a complementary manner, along with specific theoretical frameworks and models. The study’s apparent complexity is therefore intentional and justified: it enables evaluation of the clinical effectiveness of a complex intervention, while also providing valuable insight into the real-world interdisciplinary collaboration and the perceived facilitators and barriers reported by patients and healthcare professionals.^27^

### Interdisciplinary roles and the contribution of APNs

The APIS study will also explore the emerging role of APNs, positioned at the interface between clinical care and care coordination. APNs, such as clinical nurse specialists and nurse practitioners, can strengthen patients’ motivation to engage in APA by using strategies such as motivational interviewing, explaining cancer-specific benefits through personalised education and prescribing APA programmes. They can also facilitate access to appropriate resources, including community-based programmes or digital tools such as apps, thereby supporting continuity of care and behaviour change.^28^

Implementation data will highlight the complementary contributions of each professional within the interdisciplinary team, including APNs. These results will strengthen care models that support the integration of APA into patients’ daily lives, thereby promoting treatment adherence and quality of life.

### Patient-centred APA model

The APA programme proposed in the APIS study was designed to be individualised and responsive to patients’ needs, preferences and constraints. Its patient-centred design, coupled with a gradual transition toward self-directed activity, supports long-term goals of autonomy, sustainability and integration into daily life. This design aligns with current oncology recommendations advocating the personalisation of supportive care interventions and seeks to enhance adherence through a flexible, engaging framework.^29^

Importantly, patients in both the intervention and control groups are free to engage in PA independently, without restrictions. While this openness may introduce a potential bias, it is anticipated in the study protocol. A daily monitoring system, using either email or a paper logbook, has been implemented to capture all PAs performed, whether spontaneous or guided.

By accounting for these real-life conditions, the APIS study aims to provide a nuanced interpretation of its findings and strengthen the validity and applicability of the data regarding both the effectiveness of the intervention and its integration into routine care.

Moreover, a patient-researcher plays an active role in all phases of the study, contributing to its relevance, acceptability and patient-centredness and reinforcing its overall applicability.

### Limitations

Several limitations are anticipated in this study and have been carefully considered during the design phase.

First, because the intervention is behavioural, participant blinding is not feasible, which may introduce performance or reporting bias. However, this risk is partially mitigated by the inclusion of both objective clinical and paraclinical outcome measures.

Second, the use of emails or self-reported PA logs may be subject to recall or social desirability bias, even though these tools are well-validated. Their use was favoured over connected devices designed for automatic data collection. This methodological choice is based on patient feedback, which highlighted a preference for simple ones that are closer to their real-life experience.^30^

Finally, while the qualitative sample size will be guided by data saturation, transferability may be limited to similar hospital-based settings. This study represents an initial step within a type 1 hybrid design. Should the findings be positive, a subsequent phase is planned to involve community-based healthcare professionals and APA associations to support broader scaling and integration across diverse care pathways.

## Conclusion

AIMSS are the main adverse effects of AI therapy, with direct consequences on patients’ quality of life and treatment adherence. In a context where the number of patients treated with aromatase inhibitors continues to rise, the prevention and management of AIMSS have become a growing public health concern. The APIS study will assess the efficacy of an APA-based intervention for patients experiencing AIMSS. It will also provide insight into implementation processes, which may inform the development of scalable, patient-centred interventions aligned with real-world clinical constraints, ultimately enhancing equity, adherence and quality of life for breast cancer survivors.

## Supplementary material

10.1136/bmjsem-2025-003179 online supplemental file 1
