# Supplementary material for: Early adapted physical activity to prevent and manage aromatase inhibitor-induced musculoskeletal pain in breast cancer: protocol for a hybrid effectiveness-implementation randomised controlled trial (APIS)
Source: BMJ Open Sport Exerc Med. 2026 May 14;12(2):e003179. doi: 10.1136/bmjsem-2025-003179 (PMC13182384; doi:10.1136/bmjsem-2025-003179)
Supplement: online supplemental file 1 [file bmjsem-12-2-s001.docx]

**Supplementary File** Detailed description of the APA program

The APA program, delivered by the sports medicine department, was designed based on evidence. It will consist of structured exercises supervised by APA professionals aiming to achieve physiological benefits such as reducing aromatase inhibitor-induced musculoskeletal pain, improved cardiorespiratory capacity (endurance), muscle strengthening, flexibility and balance maintenance [1].

The APA program will begin, after a medical evaluation, with a 12-week supervised program within the sports medicine department. Each week, the patient will have two one-hour sessions at a sports medicine department, supervised by a physiotherapist and/or an APA instructor. Each session will consist of 30 minutes on a cycle ergometer (alternating between fundamental endurance and interval training depending on the week) followed by 30 minutes of free exercises. Free exercises refer to a personalized and supervised sequence, jointly developed by the patient and a physiotherapist or APA instructor. The patient will select the PA she wishes to engage in from a range selected by the healthcare professional (boxing, table tennis, etc.). The physiotherapist or APA instructor will then tailor the exercises based on the patient’s individual needs and functional capacities. The main objectives of these exercises will be to improve muscle strengthening, flexibility and balance maintenance. Each session will be documented as part of the implementation study.

Figure 1 shows the 12-week APA program composed of four training blocks:

- Block 1 (Weeks 1 to 3): Both sessions will involve endurance training, which will involve repeated isotonic exercises over a prolonged duration to improve aerobic capacity, performed on the cycle ergometer, followed by a series of free exercises.
- Block 2 (Weeks 4 to 6): The first session of each week will consist of interval training on the cycle ergometer, followed by free exercises, while the second session will shift to endurance training, characterized by repeated short-duration, high- to severe-intensity exercises interspersed with brief periods of lower-intensity constant-work-rate activity for active recovery and free exercises.
- Block 3 (Weeks 7 to 9): Both sessions will consist of interval training on the cycle ergometer and free exercises.
- Block 4 (Weeks 10 to 12): The first session each week will be dedicated to endurance training on the cycle ergometer and free exercises, and the second session will be interval training and free exercises.

The content of each training block, encompassing frequency, intensity, duration, type of activity (FITT), and specific modalities, will be tailored to suit each patient’s individual profile, taking into account their exercise tolerance and any PA practiced outside the APA sessions. In addition, each session will be adjusted according to the patient's heart rate, blood pressure at rest and during exercise, and perceived exertion intensity using the Borg Scale.


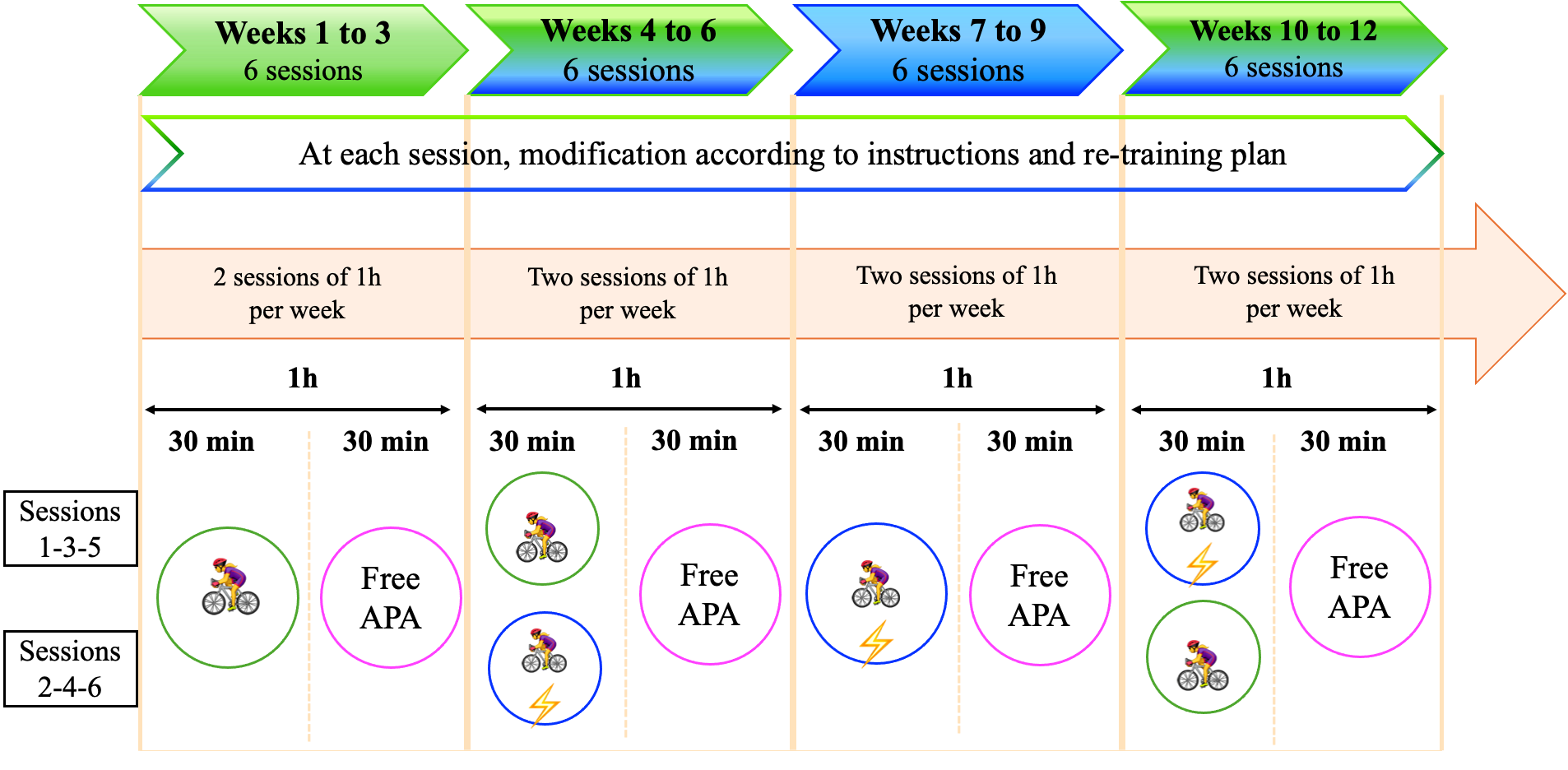


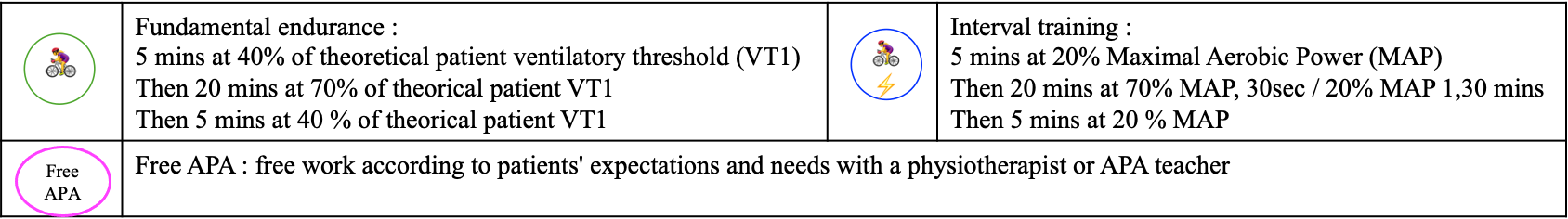


Figure 1. Progress of the APA program in the sports medicine department

The renewal or adjustment phase will begin at the end of the 12-week period with a consultation with the sports medicine physician. During this visit, the physician will evaluate the patient’s progress and the extent to which the initial goals have been achieved. Based on this evaluation, one of three options will be considered:

1) If deconditioning persists, the 12-week protocol will be renewed to reinforce the physiological effects of the APA program, which are essential for managing musculoskeletal pain induced by AI,

2) If the patient's progress meets the medical objectives but continued supervision is still necessary to maintain the regularity and effectiveness of PA, she will be referred by the sports medicine physician to a program supervised by an APA professional holding a professional certification that authorizes him to work with vulnerable populations. The recommended places include, for example, community-based PA organizations, at home, in private practice [2].

3) If the patient achieves the physician's objectives and can manage regular PA independently, she will be directed towards self-directed PA with verbal guidance.

After this period of 12 weeks, a new medical consultation will be planned and a new renewal or adjustment phase will start again until the end of the trial.

The program will gradually evolve towards independent practice depending on the patient's abilities (Figure 2).


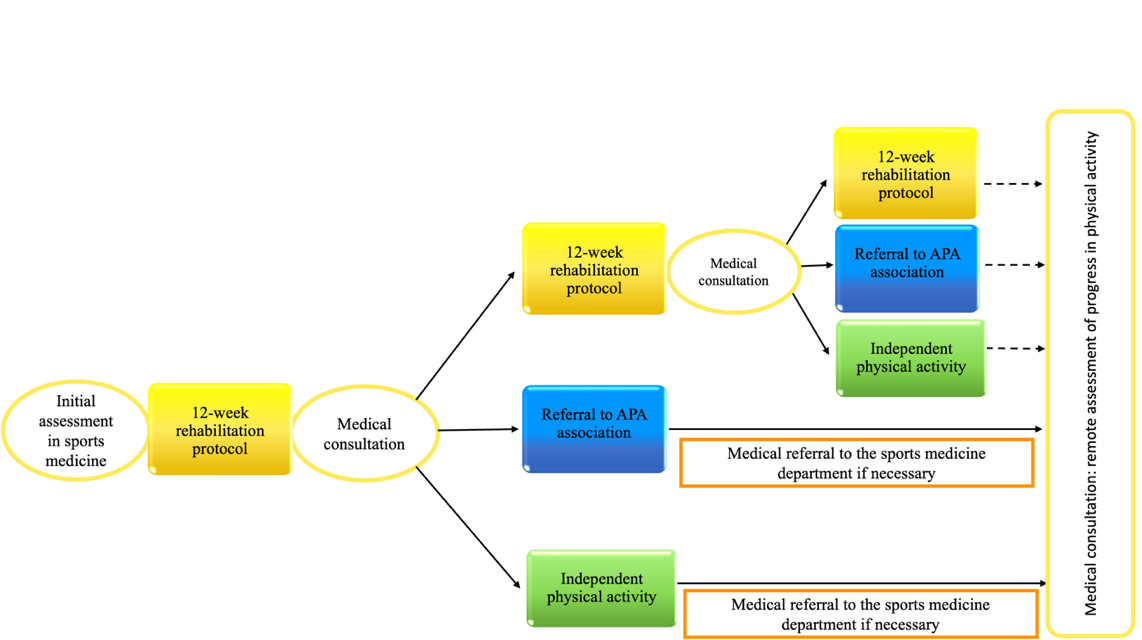


Figure 2. Progress of the personalized physical activity program

Post-operative adaptation

Following surgery, patients will temporarily reduce PA to support healing and reduce the risk of complications [1]. This recovery period is expected to last approximately one month. During this time, patients will follow home exercises, which will be taught preoperatively in individual sessions by the physiotherapist or APA instructor from the sports medicine department.

The home exercises will focus on relaxation, postural work, and painless shoulder mobility exercises, as early physical rehabilitation has been shown to improve both physical and emotional recovery after breast surgery [3]. A comprehensive booklet detailing all exercises will be provided to the patient. Before resuming the APA program, the patient will require clearance from the surgeon [1].

**Reference**

1 Campbell KL, Winters-Stone KM, Wiskemann J, *et al.* Exercise Guidelines for Cancer Survivors: Consensus Statement from International Multidisciplinary Roundtable. *Med Sci Sports Exerc*. 2019;51:2375–90. doi: 10.1249/MSS.0000000000002116

2 Association Francophone des Soins Oncologiques de Support. Activité Physique et Cancer. Paris 2024.

3 Temple-Oberle C, Shea-Budgell MA, Tan M, *et al.* Consensus Review of Optimal Perioperative Care in Breast Reconstruction: Enhanced Recovery after Surgery (ERAS) Society Recommendations. *Plast Reconstr Surg*. 2017;139:1056e–71e. doi: 10.1097/PRS.0000000000003242

**Supplementary file 3** Secondary outcomes, measurement tools and references

| **Outcome domain** | **Outcome measure** | **Data collection** | **Assessment modality** | **Reference** |
| --- | --- | --- | --- | --- |
| **Global pain^1^** | Musculoskeletal pain | Musculoskeletal pain intensity and localization | Nordic-style questionnaire^2^ | [1] |
|  | Overall pain | Self-rated overall pain | Numerical Rating Scale^2^ | [2] |
|  | Quality of life | Quality of life score | FACT-B^2^, FACT-ES^2^ | [3] |
|  | Fatigue | Fatigue score | FACIT – Fatigue^2^ | [4] |
|  | Anxiety | Anxiety and depression scores | HADS^2^ | [5] |
| **Cognitive function** | | Perceived cognitive impairments | FACT-Cog^2^ | [6] |
| **Physical condition** | Respiratory function | FEV1, PEF, VT, VC, RV, TLC, DLCO | Pulmonary function tests | [7] |
|  | Cardiac function | VO₂ peak, VO₂ max, maximal heart rate, VO₂ at VT1, cardiac output at VT1, heart rate at VT1, blood pressure, oxygen saturation | Cardiopulmonary exercise test | [8] |
|  | Cardiorespiratory endurance | Distance walked in 6 minutes | 6-minute walk test | [9] |
|  | Muscular strength | Isometric grip strength | Handgrip strength test | [10,11] |
|  | PA level | Habitual levels of PA | Marshall questionnaire^2^,  IPAQ^2^ | [12]  [13] |
| **Body composition** | Anthropometric measurements | Body weight, height, body mass index, waist circumference and 4-site skinfold | Anthropometric assessment |  |
|  | Malnutrition | Malnutrition Universal Screening Tool (MUST) score | MUST^2^ | [14] |
| **Evolution of a metabolic syndrome** | | Waist circumference | Anthropometric assessment | [15–17] |
|  |  | Blood pressure | Blood pressure measurement |  |
|  |  | Lipid profile, assessment of insulin resistance, inflammatory status | Blood tests: Lipid profile, HOMA index, C-reactive protein |  |
| **Heart rate variability (HRV)^2^** | | HRV parameters | 24-hour Holter ECG recording | [18,19] |
| **Therapeutic adherence to the AI** | | Therapeutic adherence to the AI | Girerd self- assessment questionnaire^2^ | [20] |
| **Adverse events related to PA** | | Events PA sessions | Participant self-report |  |

AI: Aromatase Inhibitor; DLCO: Diffusing Capacity of the Lung for Carbon Monoxide; FACIT-Fatigue: Functional Assessment of Chronic Illness Therapy – Fatigue; FACT-B: Functional Assessment of Cancer Therapy – Breast; FACT-Cog: Functional Assessment of Cancer Therapy - Cognitive Function; FACT-ES: Functional Assessment of Cancer Therapy - Endocrine Symptoms; FEV1: Forced Expiratory Volume in 1 Second; HADS: Hospital Anxiety and Depression Scale; IPAQ: International Physical Activity Questionnaire; MUST: Malnutrition Universal Screening Tool; PA: Physical Activity; PEF: Peak Expiratory Flow; RV: Residual Volume; TLC: Total Lung Capacity; VC: Vital Capacity; VT: Tidal Volume; VT1: ventilatory threshold 1

^1^ The evaluation of AIMSS requires a structured and multidimensional approach [21]. It will include a targeted assessment of musculoskeletal pain, a self-report of overall perceived pain and an analysis of its psychosocial impact.

^2^ Self-report questionnaires

^3^ Heart rate variability is a non-invasive indicator of autonomic cardiac regulation, reflecting the balance between sympathetic and parasympathetic activity [19]. A decrease in HRV has been associated with increased clinical vulnerability in oncology. Several studies have reported associations between altered HRV and clinical parameters such as fatigue, pain, and tumor progression [18].

**Reference**

1 Descatha A, Roquelaure Y, Chastang JF, *et al.* Validity of Nordic-style questionnaires in the surveillance of upper-limb work-related musculoskeletal disorders. *Scand J Work Environ Health*. 2007;33:58–65. doi: 10.5271/sjweh.1065

2 Jensen MP, Karoly P. Self-report scales and procedures for assessing pain in adults. *Handbook of pain assessment, 3rd ed*. New York, NY, US: The Guilford Press 2011:19–44.

3 Brady MJ, Cella DF, Mo F, *et al.* Reliability and validity of the Functional Assessment of Cancer Therapy-Breast quality-of-life instrument. *J Clin Oncol*. 1997;15:974–86. doi: 10.1200/JCO.1997.15.3.974

4 Yellen SB, Cella DF, Webster K, *et al.* Measuring fatigue and other anemia-related symptoms with the Functional Assessment of Cancer Therapy (FACT) measurement system. *J Pain Symptom Manage*. 1997;13:63–74. doi: 10.1016/s0885-3924(96)00274-6

5 Zigmond AS, Snaith RP. The hospital anxiety and depression scale. *Acta Psychiatr Scand*. 1983;67:361–70. doi: 10.1111/j.1600-0447.1983.tb09716.x

6 Joly F, Lange M, Rigal O, *et al.* French version of the Functional Assessment of Cancer Therapy-Cognitive Function (FACT-Cog) version 3. *Support Care Cancer*. 2012;20:3297–305. doi: 10.1007/s00520-012-1439-2

7 Graham BL, Steenbruggen I, Miller MR, *et al.* Standardization of Spirometry 2019 Update. An Official American Thoracic Society and European Respiratory Society Technical Statement. *Am J Respir Crit Care Med*. 2019;200:e70–88. doi: 10.1164/rccm.201908-1590ST

8 Inserm. *Activité physique : Prévention et traitement des maladies chroniques*. Paris: Éditions EDP Sciences 2019.

9 ATS Committee on Proficiency Standards for Clinical Pulmonary Function Laboratories. ATS statement: guidelines for the six-minute walk test. *Am J Respir Crit Care Med*. 2002;166:111–7. doi: 10.1164/ajrccm.166.1.at1102

10 Roberts HC, Denison HJ, Martin HJ, *et al.* A review of the measurement of grip strength in clinical and epidemiological studies: towards a standardised approach. *Age Ageing*. 2011;40:423–9. doi: 10.1093/ageing/afr051

11 Lauretani F, Russo CR, Bandinelli S, *et al.* Age-associated changes in skeletal muscles and their effect on mobility: an operational diagnosis of sarcopenia. *J Appl Physiol (1985)*. 2003;95:1851–60. doi: 10.1152/japplphysiol.00246.2003

12 Marshall AL, Smith BJ, Bauman AE, *et al.* Reliability and validity of a brief physical activity assessment for use by family doctors. *Br J Sports Med*. 2005;39:294–7; discussion 294-297. doi: 10.1136/bjsm.2004.013771

13 Craig CL, Marshall AL, Sjöström M, *et al.* International physical activity questionnaire: 12-country reliability and validity. *Med Sci Sports Exerc*. 2003;35:1381–95. doi: 10.1249/01.MSS.0000078924.61453.FB

14 Poulia K-A, Klek S, Doundoulakis I, *et al.* The two most popular malnutrition screening tools in the light of the new ESPEN consensus definition of the diagnostic criteria for malnutrition. *Clin Nutr*. 2017;36:1130–5. doi: 10.1016/j.clnu.2016.07.014

15 Alberti KGMM, Eckel RH, Grundy SM, *et al.* Harmonizing the metabolic syndrome: a joint interim statement of the International Diabetes Federation Task Force on Epidemiology and Prevention; National Heart, Lung, and Blood Institute; American Heart Association; World Heart Federation; International Atherosclerosis Society; and International Association for the Study of Obesity. *Circulation*. 2009;120:1640–5. doi: 10.1161/CIRCULATIONAHA.109.192644

16 Devaraj S, Singh U, Jialal I. Human C-reactive protein and the metabolic syndrome. *Curr Opin Lipidol*. 2009;20:182–9. doi: 10.1097/MOL.0b013e32832ac03e

17 Fahed G, Aoun L, Bou Zerdan M, *et al.* Metabolic Syndrome: Updates on Pathophysiology and Management in 2021. *Int J Mol Sci*. 2022;23:786. doi: 10.3390/ijms23020786

18 Forte G, Morelli M, Grässler B, *et al.* Decision making and heart rate variability: A systematic review. *Applied Cognitive Psychology*. 2022;36:100–10. doi: 10.1002/acp.3901

19 Lundstrom CJ, Foreman NA, Biltz G. Practices and Applications of Heart Rate Variability Monitoring in Endurance Athletes. *Int J Sports Med*. 2023;44:9–19. doi: 10.1055/a-1864-9726

20 Girerd X, Hanon O, Anagnostopoulos K, *et al.* [Assessment of antihypertensive compliance using a self-administered questionnaire: development and use in a hypertension clinic]. *Presse Med*. 2001;30:1044–8.

21 Raja SN, Carr DB, Cohen M, *et al.* The revised International Association for the Study of Pain definition of pain: concepts, challenges, and compromises. *PAIN*. 2020;161:1976. doi: 10.1097/j.pain.0000000000001939
